# Association of red blood cell distribution width-to-albumin ratio with all-cause and cardiovascular mortality in adults with hyperuricemia: A cohort study from NHANES 1999 to 2018

**DOI:** 10.1097/MD.0000000000048397

**Published:** 2026-04-17

**Authors:** Jinjun Li, Yanqing Huang, Chao Li, Quan Xun

**Affiliations:** a Department of Emergency Medicine, Liuyang People’s Hospital, Liuyang, Hunan Province, China; b Department of Pediatrics, Liuyang Hospital of Traditional Chinese Medicine, Liuyang, Hunan Province, China; c Department of Internal Medicine, Liuyang Hospital of Traditional Chinese Medicine, Liuyang, Hunan Province, China; d Department of Gastrointestinal Surgery, Liuyang People’s Hospital, Liuyang, Hunan Province, China.

**Keywords:** albumin, hyperuricemia, mortality, NHANES, red blood cell distribution width

## Abstract

The red blood cell distribution width-to-albumin ratio (RAR) reflects the combined impact of inflammation, oxidative stress, and nutritional status. However, its prognostic value for all-cause and cardiovascular disease (CVD) mortality in individuals with hyperuricemia (HUA) remains unclear. This study aimed to investigate the association between RAR and mortality risk in this particular population. Adult participants diagnosed with HUA from the National Health and Nutrition Examination Survey conducted between 1999 and 2018 were enrolled in this research. Multivariable Cox regression models and smooth curve fitting were used to investigate the associations between RAR and the risks of all-cause and CVD mortality. Kaplan–Meier survival analysis was utilized to compare survival probabilities across the different RAR quartiles (Q1–Q4). Stratified analyses were conducted to assess potential effect modification and identify subgroups at elevated risk. A total of 5735 participants with HUA were enrolled in this study. After adjusting for covariates, elevated RAR was independently associated with increased risks of both all-cause mortality (hazard ratio for Q4 vs Q1 = 3.45; 95% confidence interval: 2.58–4.61; *P* < .001) and CVD mortality (hazard ratio for Q4 vs Q1 = 3.31; 95% confidence interval: 1.90–5.76; *P* < .001). The associations exhibited nonlinear, threshold-dependent characteristics, with inflection points identified at RAR values of 3.87 for all-cause mortality and 3.71 for CVD mortality. Stratified analyses revealed significant effect modification by age for both outcomes (*P* for interaction < .001) and by gender, specifically for CVD mortality (*P* for interaction = .028). Increased RAR is independently correlated with heightened risks of all-cause and CVD mortality among American adults with HUA. These findings underscore the potential utility of RAR in stratifying mortality risk within this demographic.

## 1. Introduction

Hyperuricemia (HUA) is a widely recognized health condition, particularly prevalent in high- and middle-income countries. Recent epidemiological data indicate a concerning rise in HUA incidence, with global prevalence estimates ranging from 2.6% to 36% across different populations.^[1]^ Defined by elevated serum uric acid (SUA) concentrations, HUA is associated with various health complications, including gout, chronic kidney disease (CKD), and cardiovascular diseases (CVD).^[2,3]^ Moreover, HUA is linked to systemic inflammation, oxidative stress, and endothelial dysfunction, all of which contribute to cardiometabolic complications and increased mortality risk.^[4–7]^ Despite advances in the understanding of HUA-related comorbidities, there remains an urgent need for reliable risk assessment tools to identify individuals at increased risk of adverse outcomes.

Red blood cell distribution width (RDW), a standard measure of erythrocyte size variability, is associated with adverse outcomes in a variety of conditions, including heart failure, acute pancreatitis, and ischemic stroke.^[8–10]^ Increased RDW is thought to reflect underlying chronic inflammation, oxidative stress, and impaired erythropoiesis, all of which can contribute to endothelial dysfunction and metabolic disturbances.^[11–13]^ Serum albumin, the major plasma protein, serves as an indicator of both nutritional status and systemic inflammation. Numerous studies have reported that hypoalbuminemia is linked to poor prognosis in chronic diseases such as CVD and cancer.^[14,15]^

The red blood cell distribution width-to-albumin ratio (RAR) is a novel combined indicator that integrates these prognostic factors, thereby enhancing the ability to assess systemic inflammation and oxidative stress. Recent studies have highlighted the utility of RAR in predicting outcomes across various clinical settings, including CKD, cardiac arrest, cancer, and sepsis, suggesting its broader clinical applicability.^[16–19]^ However, to date, no studies have specifically examined RAR in populations with HUA, and the potential prognostic value of RAR in adults with HUA remains unknown. We hypothesize that a higher RAR is associated with increased mortality risk among adults with HUA. Therefore, this study aims to address this knowledge gap by investigating the association between RAR and both all-cause and CVD mortality among adults with HUA, utilizing data obtained from the National Health and Nutrition Examination Survey (NHANES) 1999 to 2018.

## 2. Materials and methods

### 2.1. Study design and population

This study employed publicly accessible, deidentified data from the NHANES, conducted by the National Center for Health Statistics under the Centers for Disease Control and Prevention. The data collection protocols for NHANES received approval from the National Center for Health Statistics Ethics Review Board, and written informed consent was obtained from all participants.^[20]^ Participant identities were maintained in strict confidentiality, with data fully anonymized and individual records identified solely by respondent sequence numbers. As this secondary analysis utilized exclusively deidentified, publicly available data devoid of protected health information, no additional ethical approval was required from our institutional review board.

In the current study, we analyzed data obtained from 10 cycles of the NHANES spanning the years 1999 to 2018, encompassing a total of 101,316 participants. Individuals under the age of 20 (n = 46,235) and those without HUA (n = 46,086) – including participants with SUA below the HUA threshold (n = 40,009) and those with missing SUA (n = 6077) – were excluded from the study. From the remaining 8995 participants diagnosed with HUA, an additional 3260 individuals were excluded. These exclusions included pregnant participants (n = 26), those with incomplete data for calculating RAR (n = 23), those without follow-up information (n = 13), and individuals with insufficient covariate data (n = 3198). Participants who were pregnant at the time of examination were excluded from the study due to the potential influence of pregnancy-related physiological changes and pregnancy-specific hypertensive disorders on SUA levels and cardiometabolic measurements. This exclusion was necessary to minimize heterogeneity and confounding factors in the association under investigation.

**Figure 1.**
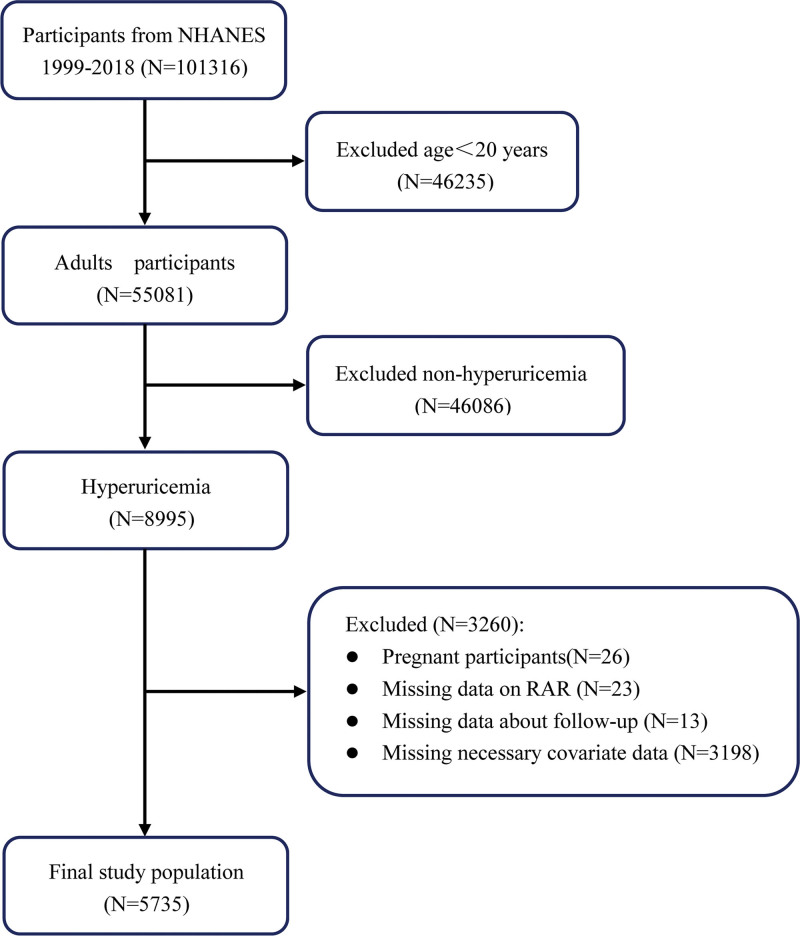
Flowchart of the study participants. NHANES = National Health and Nutrition Examination Survey, RAR = red blood cell distribution width-to-albumin ratio.

Regarding covariate missingness, we conducted a complete-case analysis and excluded participants with missing values in any covariate. The stepwise exclusions were attributable to missing data in the following variables: education (n = 13), marital status (n = 78), poverty income ratio (PIR; n = 796), body mass index (BMI; n = 176), smoking status (n = 4), drinking status (n = 2095), hypertension (n = 1), CVD (n = 28), and high-density lipoprotein cholesterol (HDL-C; n = 7). After these exclusions, the final study cohort consisted of 5735 adults diagnosed with HUA (Fig. 1).

### 2.2. Assessment of HUA and RAR

Blood specimens were measured at the NHANES Mobile Examination Centers using standardized laboratory protocols. SUA concentrations were measured using a timed-endpoint colorimetric assay. Uricase oxidizes uric acid to produce allantoin and hydrogen peroxide; the hydrogen peroxide then reacts with 4-aminoantipyrine and 3,5-dichloro-2-hydroxybenzenesulfonic acid in the presence of peroxidase to form a colored product, and the change in absorbance at 520 nm is measured to quantify SUA levels. HUA was defined as sex-specific SUA concentrations of ≥420 μmol/L (7 mg/dL) in men and ≥360 μmol/L (6 mg/dL) in women, consistent with commonly used definitions in NHANES-based studies.^[1,21–23]^

RDW was obtained from the complete blood count performed using the Beckman Coulter methodology of counting and sizing, with an automatic diluting and mixing device and a single-beam photometer for hemoglobinometry. Serum albumin was measured using the DcX 800 method, a bichromatic digital endpoint method. In this procedure, albumin forms a complex with bromocresol purple, and the change in absorbance at 600 nm is measured spectrophotometrically to determine serum albumin concentration (g/dL). RAR was determined by the equation: (RDW [%]/serum albumin [g/dL]).^[24]^

### 2.3. Mortality outcomes

The principal objectives of our research were to assess all-cause mortality and CVD mortality. The determination of survival status involved correlating data from the NHANES with records from the National Death Index, with the observation period extending until December 31, 2019. The survival duration was measured from the date of enrollment in NHANES until either the date of death or the conclusion of the follow-up period. Death causes were categorized according to the International Classification of Diseases (ICD), 10th Revision (ICD-10) codes. CVD mortality was specifically defined to encompass fatalities arising from cerebrovascular disorders (ICD-10: I60–I69) and heart disease (ICD-10: I00–I09, I11, I13, and I20–I51).^[25]^

### 2.4. Covariates

This study systematically gathered comprehensive data on demographic characteristics, lifestyle factors, health status, and laboratory indices. Demographic factors included age, gender, race, education, marital status, and PIR (low income <1.3, middle income 1.3–3.5, high income >3.5).^[26]^ Lifestyle variables included smoking status and drinking status. Smoking status was classified as never (lifetime consumption of <100 cigarettes), former (lifetime consumption of ≥100 cigarettes but not currently smoking), or current (lifetime consumption of ≥100 cigarettes and currently smoking).^[27]^ Drinking status was classified as never (fewer than 12 drinks in lifetime), low‐to-moderate (<2 drinks/day in men and <1 drink/day in women), or heavy (≥2 drinks/day in men and ≥1 drinks/day in women).^[28]^ BMI was gathered and divided into 3 ranges: <25, 25 to 30, or >30 kg/m^2^. Besides, health-related factors were evaluated, which included a history of hypertension (self-reported physician diagnosis of hypertension, prescription of antihypertensive medication, or an average systolic blood pressure ≥140 mm Hg or diastolic blood pressure ≥90 mm Hg),^[29]^ diabetes (self-reported physician diagnosis of diabetes, current use of insulin or oral glucose-lowering medications, fasting blood glucose ≥126 mg/dL, or hemoglobin A1c ≥6.5%),^[29]^ CVD (encompassing heart failure, coronary heart disease, angina, heart attack, and stroke),^[30]^ and CKD (indicated by an estimated glomerular filtration rate of <60 mL/min/1.73 m^2^ or an albumin-to-creatinine ratio ≥ 30 mg/g).^[31,32]^ In addition, levels of HDL-C and total cholesterol (TC) were also documented in the investigation.

### 2.5. Statistical analysis

In order to address the complex sampling framework of the NHANES, the present study incorporated appropriate sample weights, clustering, and stratification in the analyses to yield estimates that are representative at the national level. According to the RAR values, participants were categorized into 4 quartiles, labeled Q1 through Q4. To evaluate the normality of continuous variable distributions, the Kolmogorov–Smirnov test was applied. The results showed that none of the continuous variables followed a normal distribution (Fig. S1, Supplemental Digital Content, https://links.lww.com/MD/R710). Consequently, continuous variables in our research were represented as survey-weighted medians accompanied by interquartile ranges (IQRs), while categorical variables were depicted as survey-weighted percentages with a 95% confidence interval (CI). To compare baseline characteristics among the quartile groups of RAR, the Kruskal–Wallis test was applied for the analysis of continuous variables, while the chi-square test was utilized for categorical variables.

Weighted Cox proportional hazards models were applied to assess the correlation between RAR and all-cause as well as CVD mortality in adults with HUA. The proportional hazards assumption was evaluated using Schoenfeld residual-based diagnostics. The assumption was satisfied for RAR in both outcomes: for all-cause mortality, *P* = .219; for CVD mortality, *P* = .687. Three regression models were subsequently formulated: model 1 served as the unadjusted baseline; model 2 adjusted for age, gender, and race; and model 3 further adjusted for education, marital status, PIR, smoking, drinking, BMI, hypertension, diabetes, CVD, CKD, HDL-C, and TC. To explore potential nonlinear associations between RAR and mortality, we employed a Cox proportional hazards model augmented with cubic spline terms and conducted smooth curve estimation using penalized splines. If nonlinearity was indicated, we estimated the inflection point using a recursive algorithm and fitted a two-piecewise Cox model on either side of the inflection point. The threshold was selected by maximizing the model likelihood via a grid search (trial-and-error), and the improvement over a standard one-line Cox model was evaluated with a log-likelihood ratio test.^[6]^ Kaplan–Meier survival analyses were conducted to evaluate variations in survival probabilities over time among different quartiles of RAR, with group differences analyzed using log-rank tests. Additionally, stratified analyses were carried out to assess potential effect modification and identify subgroups at elevated risk.

Finally, to ensure the robustness of the analysis, 3 distinct sensitivity analyses were conducted. First, to tackle the potential bias introduced by absent covariate data, participants with HUA who were initially excluded due to incomplete covariate information were re-included. Missing categorical covariates were coded as a separate “missing” category. For continuous covariates, multiple imputation by chained equations was performed using the R mice package (version 3.14.0; Stef van Buuren, Utrecht, the Netherlands). Five imputed datasets were generated with 10 iterations per dataset (predictive mean matching for continuous variables). In the re-included cohort, only HDL-C (n = 10) and TC (n = 7) had missing values and were imputed. Each imputed dataset was analyzed using the same survey-weighted Cox models, and estimates were pooled using Rubin’s rules. Second, as a sensitivity analysis to assess potential reverse causation, we repeated the analyses after excluding participants who died within the first 2 years of follow-up. Lastly, individuals aged 80 years and above were eliminated in order to lessen the likelihood of survival bias related to this very elderly cohort. Statistical analyses were performed using R (version 4.2.3; R Foundation for Statistical Computing, Vienna, Austria) and EmpowerStats (version 5.2; X&Y Solutions, Inc., Boston), with a two-sided *P* < .05 regarded as statistically significant.

## 3. Results

### 3.1. Baseline characteristics

As depicted in Table 1, the study cohort was categorized into 4 groups based on the RAR quartiles: Q1 (2.173–2.826), Q2 (2.829–3.073), Q3 (3.075–3.375), and Q4 (3.377–8.087). The final study cohort consisted of 5735 participants, with a median age of 49.00 years (IQR: 34.00–63.00). Among these individuals, 59.49% were male, while 40.51% were female. A significant portion of the population identified as non-Hispanic White (72.27%), with 59.39% having attained education beyond high school, 61.5% being married or cohabiting with a partner, and 44.62% possessing a high income. The median BMI was recorded at 30.60 kg/m^2^ (IQR: 26.93–35.71), with 54.49% of participants classified as obese (BMI ≥ 30 kg/m^2^). Regarding lifestyle factors, 19.10% of participants were current smokers, and 45.19% were heavy drinkers. Prevalent clinical conditions within the cohort included hypertension (54.22%), diabetes (15.15%), CVD (11.31%), and CKD (23.9%).

**Table 1:** Baseline characteristics of the study population.

Variables	Overall	RAR				
		Quartile 1 (2.173–2.826)	Quartile 2 (2.829–3.073)	Quartile 3 (3.075–3.375)	Quartile 4 (3.377–8.087)	*P*
Participants	5735	1435	1435	1432	1433	
Age (yr)	49.00 (34.00, 63.00)	40.00 (28.00, 53.00)	48.00 (35.00, 62.00)	54.00 (40.00, 68.00)	57.00 (44.00, 70.00)	<.001
Age group						<.001
20–59	69.62 (68.09, 71.11)	84.70 (82.45, 86.71)	70.00 (67.31, 72.55)	60.10 (56.82, 63.28)	55.83 (52.11, 59.48)	
≥60	30.38 (28.89, 31.91)	15.30 (13.29, 17.55)	30.00 (27.45, 32.69)	39.90 (36.72, 43.18)	44.17 (40.52, 47.89)	
Gender						<.001
Male	59.49 (57.94, 61.03)	78.52 (75.68, 81.11)	62.04 (58.48, 65.47)	48.79 (45.11, 52.48)	37.55 (34.69, 40.51)	
Female	40.51 (38.97, 42.06)	21.48 (18.89, 24.32)	37.96 (34.53, 41.52)	51.21 (47.52, 54.89)	62.45 (59.49, 65.31)	
Race						<.001
Mexican American	5.85 (4.92, 6.96)	6.65 (5.38, 8.19)	6.07 (4.80, 7.65)	5.60 (4.33, 7.21)	4.55 (3.37, 6.12)	
Non-Hispanic White	72.27 (69.99, 74.44)	78.61 (76.01, 81.00)	74.72 (71.47, 77.72)	72.24 (68.85, 75.40)	58.35 (54.28, 62.32)	
Non-Hispanic Black	11.25 (9.92, 12.74)	4.12 (3.31, 5.11)	7.83 (6.55, 9.33)	12.52 (10.64, 14.68)	26.30 (23.04, 29.85)	
Other races	10.63 (9.43, 11.95)	10.63 (8.69, 12.93)	11.37 (9.47, 13.60)	9.64 (7.86, 11.77)	10.80 (8.96, 12.96)	
Education						<.001
Under high school	14.64 (13.62, 15.71)	11.45 (9.88, 13.24)	14.84 (12.81, 17.14)	15.37 (13.28, 17.71)	18.73 (16.69, 20.96)	
High school or equivalent	25.98 (24.43, 27.58)	26.68 (23.93, 29.62)	24.09 (21.22, 27.22)	26.41 (23.48, 29.55)	26.91 (23.98, 30.05)	
Above high school	59.39 (57.48, 61.27)	61.87 (58.57, 65.07)	61.07 (57.68, 64.35)	58.23 (54.72, 61.65)	54.36 (51.07, 57.62)	
Marital status						<.001
Married/living with partner	61.5 (59.50, 63.46)	63.52 (60.00, 66.90)	67.76 (64.55, 70.80)	58.36 (54.75, 61.88)	53.27 (49.63, 56.87)	
Widowed/divorced/separated	20.52 (19.26, 21.84)	13.15 (11.21, 15.37)	17.14 (15.06, 19.45)	25.77 (23.14, 28.59)	31.02 (27.47, 34.80)	
Never married	17.98 (16.48, 19.58)	23.33 (20.42, 26.53)	15.10 (12.85, 17.66)	15.87 (13.17, 19.00)	15.71 (13.16, 18.66)	
PIR						<.001
Low income	18.82 (17.48, 20.23)	14.13 (12.21, 16.30)	18.04 (15.95, 20.33)	19.61 (17.40, 22.02)	26.71 (24.16, 29.42)	
Middle income	36.57 (34.77, 38.41)	34.80 (31.90, 37.81)	34.91 (31.54, 38.44)	38.07 (35.03, 41.21)	39.96 (36.62, 43.39)	
High income	44.62 (42.35, 46.90)	51.07 (47.60, 54.54)	47.05 (43.52, 50.61)	42.32 (38.87, 45.84)	33.33 (29.68, 37.19)	
BMI (kg/m^2^)	30.60 (26.93, 35.71)	28.80 (25.90, 32.04)	30.65 (26.94, 35.42)	31.90 (27.90, 37.50)	34.30 (28.90, 41.40)	<.001
BMI category						<.001
<25	13.5 (12.46, 14.62)	18.31 (15.84, 21.07)	13.01 (11.15, 15.14)	11.54 (9.36, 14.15)	8.62 (6.94, 10.65)	
25–30	32.01 (30.23, 33.83)	42.16 (39.03, 45.36)	32.46 (28.89, 36.25)	27.15 (24.31, 30.20)	20.50 (17.64, 23.68)	
≥30	54.49 (52.58, 56.39)	39.53 (36.28, 42.87)	54.52 (51.06, 57.94)	61.31 (57.68, 64.82)	70.89 (67.18, 74.34)	
Smoking status						.4514
Never	51.96 (50.10, 53.81)	52.17 (48.79, 55.53)	52.59 (48.69, 56.47)	50.72 (46.99, 54.45)	52.26 (48.47, 56.03)	
Former	28.94 (27.24, 30.70)	27.26 (24.64, 30.04)	29.10 (25.74, 32.72)	29.37 (26.04, 32.94)	30.97 (27.11, 35.11)	
Current	19.10 (17.68, 20.60)	20.58 (18.07, 23.33)	18.30 (16.12, 20.71)	19.91 (17.14, 22.99)	16.77 (14.45, 19.37)	
Drinking status						<.001
Never	13.69 (12.10, 15.44)	8.48 (6.68, 10.70)	13.04 (10.36, 16.28)	16.75 (14.34, 19.48)	19.46 (16.77, 22.46)	
Low-to-moderate	41.12 (39.04, 43.24)	39.51 (36.32, 42.79)	41.88 (38.01, 45.85)	41.79 (37.83, 45.85)	41.94 (38.82, 45.13)	
Heavy	45.19 (43.21, 47.19)	52.02 (48.62, 55.39)	45.08 (41.52, 48.70)	41.46 (37.55, 45.49)	38.60 (35.09, 42.24)	
Hypertension						<.001
No	45.78 (43.88, 47.68)	59.31 (56.03, 62.51)	46.52 (43.49, 49.58)	39.21 (35.69, 42.85)	30.37 (27.05, 33.91)	
Yes	54.22 (52.32, 56.12)	40.69 (37.49, 43.97)	53.48 (50.42, 56.51)	60.79 (57.15, 64.31)	69.63 (66.09, 72.95)	
Diabetes						<.001
No	84.85 (83.69, 85.94)	94.61 (93.22, 95.72)	87.40 (85.12, 89.38)	81.50 (78.76, 83.95)	69.22 (66.09, 72.18)	
Yes	15.15 (14.06, 16.31)	5.39 (4.28, 6.78)	12.60 (10.62, 14.88)	18.50 (16.05, 21.24)	30.78 (27.82, 33.91)	
CVD						<.001
No	88.69 (87.58, 89.72)	94.02 (92.52, 95.23)	91.69 (89.59, 93.39)	85.90 (83.51, 87.99)	79.12 (76.00, 81.94)	
Yes	11.31 (10.28, 12.42)	5.98 (4.77, 7.48)	8.31 (6.61, 10.41)	14.10 (12.01, 16.49)	20.88 (18.06, 24.00)	
CKD						<.001
No	76.1 (74.73, 77.41)	89.29 (87.11, 91.15)	78.82 (76.07, 81.34)	68.46 (65.50, 71.28)	59.80 (56.31, 63.18)	
Yes	23.9 (22.59, 25.27)	10.71 (8.85, 12.89)	21.18 (18.66, 23.93)	31.54 (28.72, 34.50)	40.20 (36.82, 43.69)	
HDL-C (mg/dL)	46.00 (39.00, 57.00)	45.00 (38.00, 55.00)	45.00 (38.00, 56.00)	47.00 (40.00, 57.00)	48.00 (40.00, 59.00)	<.001
TC (mg/dL)	201.00 (174.00, 229.00)	207.00 (180.00, 236.00)	202.00 (176.00, 230.00)	197.00 (174.00, 224.00)	191.00 (165.00, 222.00)	<.001

BMI = body mass index, CKD = chronic kidney disease, CVD = cardiovascular disease, HDL-C = high-density lipoprotein cholesterol, PIR = family poverty income ratio, RAR = red blood cell distribution width-to-albumin ratio, TC = total cholesterol.

Participants classified within the upper quartiles of the RAR exhibited a tendency towards being older, predominantly female, and of non-Hispanic Black ethnicity. Marital status exhibited significant variation across quartiles, with a decreasing proportion of married or cohabiting individuals and an increasing proportion of those who were widowed, divorced, or separated. Metabolic profiles showed progressive deterioration across the quartiles, characterized by higher incidences of obesity and comorbidities such as hypertension, diabetes, CVD, and CKD. Furthermore, participants situated in the higher quartiles displayed higher values of HDL-C and diminished values of TC. Notably, no significant differences were identified in smoking status across the quartiles (*P* = .451).

### 3.2. Association between RAR and mortality

**Figure 2.**
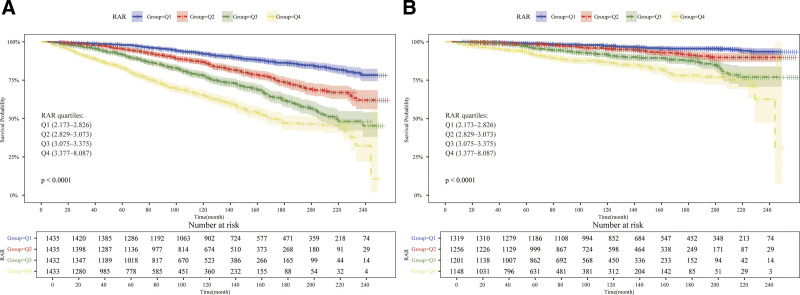
Kaplan–Meier survival curves for all-cause and CVD mortality based on RAR quartiles in adults with hyperuricemia. (A) All-cause mortality. (B) CVD mortality. *P* values were calculated using the log-rank test. The x-axis indicates the follow-up time in months, and the y-axis indicates the cumulative survival probability. CVD = cardiovascular disease, RAR = red blood cell distribution width-to-albumin ratio.

The median follow-up duration was 108 months, during which 1133 deaths were documented, including 322 deaths due to CVD. The Kaplan–Meier survival analysis demonstrated a notable correlation between increased RAR values and a heightened risk of mortality due to all causes as well as CVD among individuals with HUA (*P* < .001). The survival probability remained significantly lower in the Q4 group in comparison to the Q1 group across all evaluated time intervals (Fig. 2).

Weighted Cox regression analyses demonstrated notable correlations between the risk of mortality and the RAR across increasingly adjusted models (Table 2). The proportional hazards assumption was not violated for RAR (all-cause mortality: *P* = .219; CVD mortality: *P* = .687). In terms of all-cause mortality, elevated RAR values consistently demonstrated a greater risk in all models: model 1 (hazard ratio [HR] 3.04, 95% CI: 2.61–3.53), model 2 (HR 2.48, 95% CI: 2.04–3.01), and model 3 (HR 2.15, 95% CI: 1.81–2.56). Similarly, a positive correlation between RAR levels and CVD mortality was identified in all models, with HR of 3.24 (95% CI: 2.70–3.88) in model 1, 2.49 (95% CI: 1.99–3.13) in model 2, and 2.15 (95% CI: 1.65–2.80) in model 3. Additionally, participants in the Q4 group exhibited a markedly increased risk for both all-cause (HR 3.45, 95% CI: 2.58–4.61) and CVD mortality (HR 3.31, 95% CI: 1.90–5.76) compared with those in the Q1 group. All trend analyses across the models reached statistically significant (all *P* for trend < .001).

**Table 2:** Association between the red blood cell distribution width-to-albumin ratio and all-cause and cardiovascular mortality.

Variables	Model 1		Model 2		Model 3	
	HR (95% CI)	*P*	HR (95% CI)	*P*	HR (95% CI)	*P*
All-cause mortality						
RAR	3.04 (2.61–3.53)	<.001	2.48 (2.04– 3.01)	<.001	2.15 (1.81– 2.56)	<.001
RAR quartiles						
Q1	Reference		Reference		Reference	
Q2	2.14 (1.69–2.72)	<.001	1.56 (1.25–1.93)	<.001	1.47 (1.16–1.86)	.001
Q3	3.71 (2.91–4.74)	<.001	2.32 (1.80–2.99)	<.001	1.95 (1.47–2.60)	<.001
Q4	6.91 (5.37–8.89)	<.001	4.54 (3.47–5.96)	<.001	3.45 (2.58–4.61)	<.001
*P* for trend		<.001		<.001		<.001
CVD mortality						
RAR	3.24 (2.70–3.88)	<.001	2.49 (1.99–3.13)	<.001	2.15 (1.65–2.80)	<.001
RAR quartiles						
Q1	Reference		Reference		Reference	
Q2	1.86 (1.17–2.97)	.009	1.36 (0.84–2.23)	.215	1.33 (0.79–2.25)	.288
Q3	4.29 (2.74–6.72)	<.001	2.57 (1.57–4.22)	<.001	2.00 (1.15–3.48)	.014
Q4	7.58 (4.98–11.55)	<.001	4.97 (3.03–8.16)	<.001	3.31 (1.90–5.76)	<.001
*P* for trend		<.001		<.001		<.001

Model 1: non-adjusted.

Model 2: adjusted for age, gender, and race.

Model 3: adjusted for age, gender, race, education, marital status, PIR, smoking status, drinking status, BMI category, hypertension, diabetes, CVD, CKD, HDL-C, and TC.

BMI = body mass index, CI = confidence interval, CKD = chronic kidney disease, CVD = cardiovascular disease, HDL-C = high-density lipoprotein cholesterol, HR = hazard ratio, PIR = family poverty income ratio, RAR = red blood cell distribution width-to-albumin ratio, TC = total cholesterol.

### 3.3. Dose–response relationship between RAR and mortality

**Figure 3.**
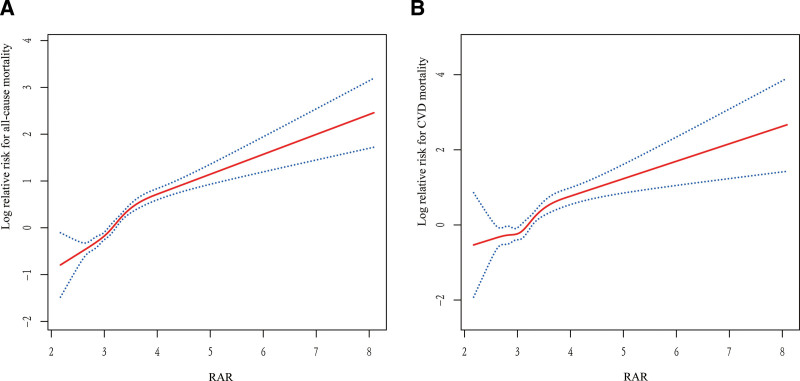
Dose–response relationships between RAR and all-cause and CVD mortality. (A) All-cause mortality. (B) CVD mortality. The solid line and dashed line represent the estimated values and their corresponding 95% confidence intervals. CVD = cardiovascular disease, RAR = red blood cell distribution width-to-albumin ratio.

After adjustment for age, gender, race, education, marital status, PIR, smoking status, drinking status, BMI category, hypertension, diabetes, CVD, CKD, HDL-C, and TC, the results of the fully adjusted smooth curve fitting analysis showed a significant positive association between RAR and mortality (Fig. 3). Specifically, higher levels of RAR were associated with an increased risk of both all-cause mortality and CVD mortality, with the log relative risk rising steadily as RAR increased.

Threshold effect analysis identified critical inflection points for RAR at 3.87 for overall mortality and 3.71 for CVD mortality (Table 3). For RAR levels below these thresholds, the HRs for all-cause and CVD mortality were 2.81 (95% CI: 2.33–3.40) and 2.91 (95% CI: 1.94–4.35), respectively, indicating a substantial escalation in risk. Conversely, HRs above these thresholds were lower but still statistically significant (all-cause mortality: HR 1.39, 95% CI: 1.12–1.71; CVD mortality: HR 1.57, 95% CI: 1.14–2.14). Likelihood ratio tests further verified a statistically significant difference in risk slopes on either side of the inflection points for all-cause mortality (*P* < .001) and CVD mortality (*P* = .034). The results imply a threshold-dependent, nonlinear relationship between RAR and mortality among individuals with HUA.

**Table 3:** Threshold effect analysis of the red blood cell distribution width-to-albumin ratio for all-cause and cardiovascular mortality.

Models	All-cause mortality		CVD mortality	
	HR (95% CI)	*P*	HR (95% CI)	*P*
Model 1				
Linear effect	1.96 (1.78–2.17)	<.001	1.98 (1.65–2.37)	<.001
Model 2				
Inflection point (K)	3.87		3.71	
RAR < K	2.81 (2.33–3.40)	<.001	2.91 (1.94–4.35)	<.001
RAR > K	1.39 (1.12–1.71)	.0025	1.57 (1.14–2.14)	.0051
*P* for log-likelihood ratio	<.001		.034	

CI = confidence interval, CVD = cardiovascular disease, HR = hazard ratio, RAR = red blood cell distribution width-to-albumin ratio.

### 3.4. Stratified analyses

The positive association between elevated RAR and all-cause mortality remained robust in all subgroups defined by age, gender, BMI categories, smoking status, drinking status, hypertension, diabetes, and CKD (all *P* < .001). The interaction test indicated that the correlation between RAR and the likelihood of both all-cause and CVD mortality in adults with HUA was markedly affected by age (*P* for interaction < .001). Specifically, younger participants (aged 20–59 years) exhibited higher mortality risks compared with those aged 60 years and above. Additionally, the impact of RAR on CVD mortality was significantly affected by gender (*P* for interaction = .028), with females demonstrating a higher risk. A noteworthy interaction was also observed between hypertension and RAR for overall mortality (*P* for interaction = .037). By contrast, there were no meaningful interactions related to BMI, smoking status, drinking status, diabetes, or CKD (Table 4).

**Table 4:** Subgroup analyses of the association between the red blood cell distribution width-to-albumin ratio and all-cause and cardiovascular mortality.

Variables	All-cause mortality			CVD mortality		
	HR (95% CI)	*P*	*P* for interaction	HR (95% CI)	*P*	*P* for interaction
Age			<.001			<.001
20–59	3.02 (2.31–3.94)	<.001		4.88 (2.95–8.07)	<.001	
≥60	1.87 (1.56–2.25)	<.001		1.78 (1.32–2.41)	<.001	
Gender			.244			.028
Male	1.92 (1.56–2.36)	<.001		1.93 (1.4–2.66)	<.001	
Female	2.65 (2.04–3.44)	<.001		3.01 (1.86–4.89)	<.001	
BMI category			.061			.271
<25	2.7 (1.98–3.69)	<.001		5.92 (2.53–13.87)	<.001	
25–30	2.53 (1.89–3.37)	<.001		1.85 (1.05–3.27)	.032	
≥30	2.05 (1.66–2.55)	<.001		2.28 (1.72–3.02)	<.001	
Smoking status			.476			.415
Never	2.27 (1.68–3.07)	<.001		2.07 (1.35–3.17)	.001	
Former	2.22 (1.64–3)	<.001		2.24 (1.46–3.41)	<.001	
Current	1.99 (1.51–2.63)	<.001		2.66 (1.5–4.72)	.001	
Drinking status			.549			.153
Never	2.99 (2.18–4.1)	<.001		4.06 (2.28–7.22)	<.001	
Low-to-moderate	2.09 (1.65–2.66)	<.001		2.31 (1.64–3.27)	<.001	
Heavy	2.09 (1.5–2.92)	<.001		1.74 (1.1–2.74)	.017	
Hypertension			.037			.989
No	3.06 (2.02–4.62)	<.001		1.62 (0.72–3.66)	.242	
Yes	2.03 (1.69–2.44)	<.001		2.15 (1.61–2.87)	<.001	
Diabetes			.091			.896
No	2.33 (1.83–2.97)	<.001		2.1 (1.34–3.3)	.001	
Yes	2.09 (1.63–2.69)	<.001		2.61 (1.72–3.96)	<.001	
CKD			.084			.631
No	2.33 (1.78–3.05)	<.001		2.58 (1.61–4.12)	<.001	
Yes	2.04 (1.65–2.52)	<.001		2.02 (1.46–2.8)	<.001	

BMI = body mass index, CI = confidence interval, CKD = chronic kidney disease, CVD = cardiovascular disease, HR = hazard ratio.

### 3.5. Sensitivity analysis

The primary results remained stable in the 3 sensitivity analyses. After re-including individuals who were initially excluded from the study due to missing covariate data, the relationship between elevated RAR and both all-cause and CVD mortality persisted consistently (Table S1, Supplemental Digital Content, https://links.lww.com/MD/R710). Additionally, consistent findings were noted upon the exclusion of individuals who expired within the initial 2 years of follow-up (Table S2, Supplemental Digital Content, https://links.lww.com/MD/R710), as well as those aged 80 years and above (Table S3, Supplemental Digital Content, https://links.lww.com/MD/R710).

## 4. Discussion

Our study suggests that higher RAR levels are independently associated with increased all-cause and CVD mortality among adults with HUA. Over a median follow-up period of 108 months, individuals in the highest RAR quartile exhibited a 3.45-fold increased risk of all-cause mortality and a 3.31-fold increased risk of CVD mortality compared with those in the lowest quartile, after multivariable adjustment. These findings are consistent with prior reports that elevated RAR is associated with adverse outcomes in conditions such as type 2 diabetes, heart failure, acute myocardial infarction, and chronic obstructive pulmonary disease.^[33–36]^ Our study extends this evidence by showing an association between RAR and both all-cause and CVD mortality in adults with HUA. From a clinical perspective, RAR is derived from RDW and serum albumin – 2 widely available, low-cost laboratory indices routinely measured in clinical practice – thereby offering a pragmatic marker that may help identify higher-risk individuals with HUA who could benefit from closer follow-up and intensified management of cardiometabolic risk factors. Importantly, RAR should be interpreted as a risk marker rather than a causal factor or therapeutic target, and whether it provides incremental prognostic information beyond established risk factors requires further validation.

Additionally, we observed a nonlinear, threshold-dependent relationship between RAR and mortality, with inflection points at 3.87 for all-cause mortality and 3.71 for CVD mortality. In fully adjusted piecewise models, the estimated association was stronger below the inflection points (all-cause mortality: HR 2.81, 95% CI: 2.33–3.40; CVD mortality: HR 2.91, 95% CI: 1.94–4.35) and remained statistically significant but attenuated above the thresholds (all-cause mortality: HR 1.39, 95% CI: 1.12–1.71; CVD mortality: HR 1.57, 95% CI: 1.14–2.14); likelihood ratio tests supported different slopes across the inflection points. Clinically, these findings suggest that mortality risk increases more prominently as RAR rises to approximately 3.7 to 3.9, after which further increases may be associated with a less pronounced rise in risk. However, because these cut points were derived from the current dataset and may vary by population characteristics, laboratory methods, comorbidity burden, and baseline nutritional or inflammatory status, they should be considered exploratory and not used as definitive clinical decision thresholds until replicated in independent cohorts and prospective studies. Future research should evaluate whether adding RAR improves risk prediction beyond established risk factors, and whether repeated (time-updated) RAR measurements better capture risk trajectories than a single baseline assessment.

The observed association between elevated RAR and mortality in individuals with HUA may be attributable to the combined effects of HUA, systemic inflammation, and oxidative stress. Monosodium urate crystals can activate the NOD-like receptor family pyrin domain containing 3 inflammasome, leading to the release of interleukin-1β and the development of chronic inflammation.^[37,38]^ This inflammatory environment has been shown to disrupt erythropoiesis and reduce erythrocyte deformability, thereby increasing RDW.^[39]^ Inflammatory cytokines such as interleukin-1β, interleukin-6, and tumor necrosis factor-α may further impair bone marrow function and exacerbate anisocytosis.^[40,41]^ Additionally, inflammation is known to suppress hepatic albumin synthesis,^[42]^ and lower serum albumin levels may compromise the body’s antioxidant defenses, promote reactive oxygen species generation,^[43,44]^ and contribute to atherosclerosis progression.^[45]^ Collectively, these mechanisms may explain why RAR, which integrates information about both systemic inflammation (via RDW) and nutritional/oxidative status (via albumin), is associated with increased mortality risk in the HUA population. Notably, some experimental studies suggest that uric acid-lowering therapies can reduce NOD-like receptor family pyrin domain containing 3 inflammasome activation and improve endothelial function,^[46]^ pointing toward potential avenues for intervention. However, further mechanistic and longitudinal studies are needed to confirm these pathways and to determine whether targeting these processes can reduce RAR-associated mortality risk in individuals with HUA.

In our subgroup analysis, age and gender emerged as significant effect modifiers. Notably, younger adults (20–59 years) exhibited a stronger association between higher RAR and increased risks of all-cause and CVD mortality, compared with those aged 60 years and above. This heightened susceptibility in younger individuals may be due to longer cumulative exposure to subclinical metabolic disturbances and latent vascular injury, while in older adults, the effect of RAR could be diluted by a higher burden of competing comorbidities.^[47,48]^ We also observed sex-specific differences in CVD mortality, with women exhibiting higher risks related to elevated RAR. One possible explanation is the decline in estrogen levels after menopause, as estrogen exerts cardiovascular protection through antioxidative and endothelial effects.^[49,50]^ Postmenopausal estrogen deficiency is also associated with adverse changes in cardiovascular risk profiles, including increased blood pressure, dyslipidemia, and heightened sympathetic activity, all of which may contribute to the excess CVD risk among women.^[51]^ These findings highlight the need for further studies to clarify the biological mechanisms underlying the observed age- and gender-related differences in the associations between RAR and mortality in individuals with HUA.

This study has several notable strengths. First, it is the first to evaluate the association between RAR and mortality risk specifically in American adults with HUA. The use of a large, nationally representative cohort enhances the generalizability of the findings. Rigorous adjustments for a wide array of confounding variables, along with comprehensive sensitivity analyses, further strengthen the validity of the results. Additionally, the identification of nonlinear, threshold-dependent relationships, including specific inflection points for RAR in relation to both all-cause and CVD mortality, provides important new insights.

However, several limitations should be acknowledged. First, the observational design precludes causal inference. Second, although extensive covariates were adjusted for, residual confounding may persist due to unmeasured or incompletely measured factors, such as dietary purine intake, overall dietary patterns, physical activity, inflammatory status, socioeconomic factors, and healthcare access. Third, the use of medications such as systemic steroids, diuretics, and urate-lowering drugs was not explicitly considered in the analysis. Such medication use could be associated with both RAR and mortality risk; consequently, the observed associations may have been attenuated toward the null, potentially leading to an underestimation of the association between RAR and mortality. In addition, RAR and other covariates were assessed only at baseline, and longitudinal changes during follow-up were not captured. Finally, because the study was limited to US adults with HUA, the generalizability of these findings to other populations may be limited.

In summary, elevated RAR was independently associated with an increased risk of all-cause and CVD mortality among American adults with HUA. A nonlinear, threshold-dependent relationship was observed between RAR and mortality risk. These findings suggest that RAR may be a useful indicator for identifying individuals at higher risk within this population. Future prospective cohort studies are warranted to validate these findings and clarify their clinical applicability.

## Acknowledgments

We thank the NHANES team for data collection and follow-up, and all participants for their contribution.

## Author contributions

**Conceptualization:** Jinjun Li, Yanqing Huang.

**Data curation:** Jinjun Li, Yanqing Huang.

**Formal analysis:** Jinjun Li, Chao Li.

**Software:** Jinjun Li, Chao Li.

**Supervision:** Quan Xun.

**Writing – original draft:** Jinjun Li.

**Writing – review & editing:** Quan Xun.

## Supplementary Material


