# Supplementary material for: Association of red blood cell distribution width-to-albumin ratio with all-cause and cardiovascular mortality in adults with hyperuricemia: A cohort study from NHANES 1999 to 2018
Source: Medicine (Baltimore). 2026 Apr 17;105(16):e48397. doi: 10.1097/MD.0000000000048397 (PMC13095257; doi:10.1097/MD.0000000000048397)
Supplement: Supplementary file 1 [file medi-105-e48397-s001.pdf]

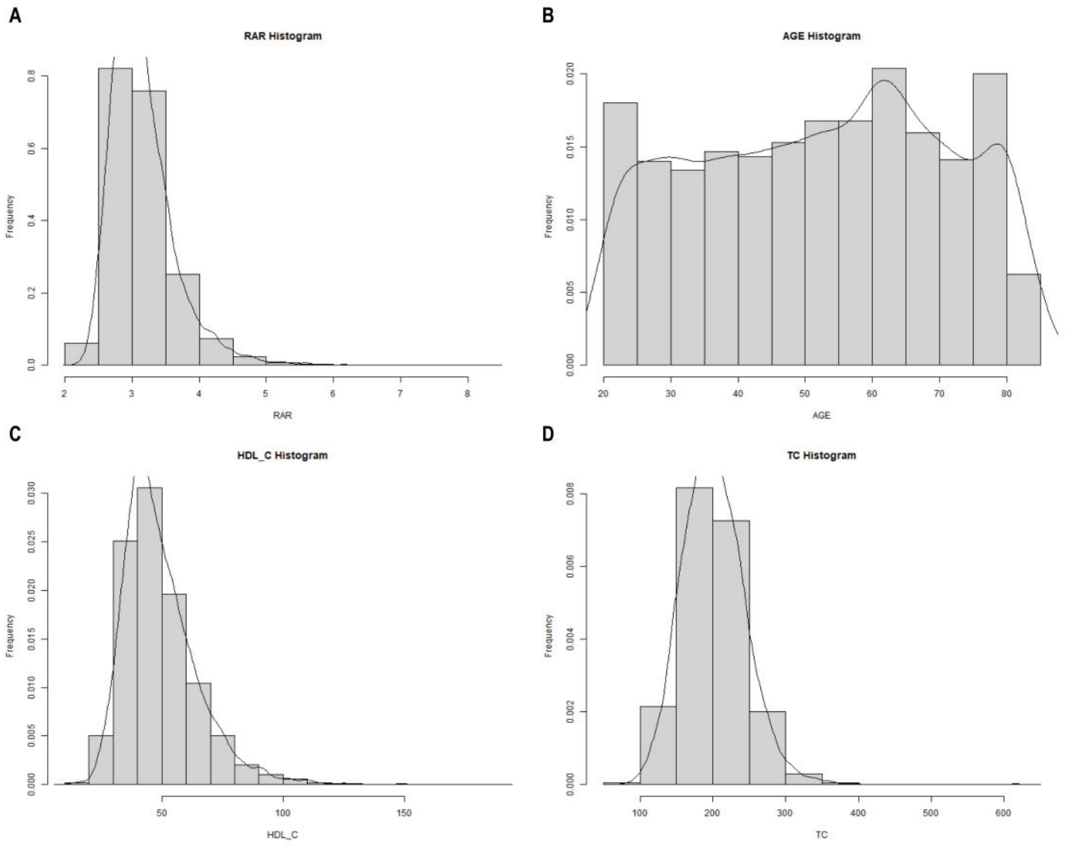

Supplementary Figure S1. Distribution of the continuous variables in this study.

RAR = red blood cell distribution width-to-albumin ratio; AGE = age; HDL-C = high-density lipoprotein cholesterol; TC = total cholesterol.

Supplementary Table S1. Association between the red blood cell distribution width-to-albumin ratio and all-cause and cardiovascular mortality in adults with hyperuricemia after re-including participants with missing covariate data.

Variables	Model 1		Model 2		Model 3	
	HR(95% CI)	*P*	HR(95% CI)	*P*	HR(95% CI)	*P*
**All-cause mortality**						
RAR	2.42(2.08,2.81)	< .001	2.16(1.87,2.49)	< .001	1.81(1.59,2.05)	< .001
RAR quartiles						
Q1	reference		reference		reference	
Q2	1.97(1.66,2.33)	< .001	1.43(1.22,1.69)	< .001	1.38(1.16,1.65)	< .001
Q3	3.25(2.73,3.87)	< .001	2.05(1.71,2.47)	< .001	1.87(1.55,2.25)	< .001
Q4	6.47(5.41,7.73)	< .001	4.21(3.48,5.08)	< .001	3.03(2.49,3.70)	< .001
P for trend		< .001		< .001		< .001
**CVD mortality**						
RAR	2.90(2.31,3.63)	< .001	2.69(2.27,3.18)	< .001	2.19(1.82,2.64)	< .001
RAR quartiles						
Q1	reference		reference		reference	
Q2	2.20(1.52,3.19)	< .001	1.57(1.08,2.30)	.019	1.45(0.99,2.12)	.052
Q3	4.23(2.95,6.06)	< .001	2.52(1.71,3.71)	< .001	2.11(1.41,3.16)	< .001
Q4	8.92(6.3,12.64)	< .001	5.69(3.84,8.45)	< .001	3.62(2.35,5.60)	< .001
P for trend		< .001		< .001		< .001

Model 1: non-adjusted;

Model 2: adjusted for age, gender, race;

Model 3: adjusted for age, gender, race, educational level, marital status, PIR, smoking status, drinking status, BMI category, hypertension, diabetes, CVD, CKD, HDL-C, and TC.

RAR = red blood cell distribution width-to-albumin ratio; HUA = hyperuricemia; PIR = family poverty income ratio; BMI = body mass index; CVD = cardiovascular disease; CKD = chronic kidney disease; HDL-C = high-density lipoprotein cholesterol; TC = total cholesterol; HR = hazard ratio; CI = confidence interval.

Supplementary Table S2. Association between the red blood cell distribution width-to-albumin ratio and all-cause and cardiovascular mortality in adults with hyperuricemia after excluding participants who died within 2 years of follow-up.

Variables	Model 1		Model 2		Model 3	
	HR(95% CI)	*P*	HR(95% CI)	*P*	HR(95% CI)	*P*
**All-cause mortality**						
RAR	2.93(2.51,3.42)	< .001	2.31(1.90,2.81)	< .001	1.99(1.66,2.40)	< .001
RAR quartiles						
Q1	reference		reference		reference	
Q2	2.18(1.71,2.79)	< .001	1.55(1.24,1.95)	< .001	1.47(1.14,1.89)	.003
Q3	3.52(2.73,4.53)	< .001	2.16(1.65,2.83)	< .001	1.88(1.39,2.53)	< .001
Q4	6.52(4.95,8.58)	< .001	4.20(3.12,5.65)	< .001	3.29(2.40,4.52)	< .001
P for trend		< .001		< .001		< .001
**CVD mortality**						
RAR	3.06(2.53,3.71)	< .001	2.31(1.81,2.95)	< .001	1.97(1.47,2.63)	< .001
RAR quartiles						
Q1	reference		reference		reference	
Q2	1.83(1.15,2.91)	.011	1.32(0.82,2.12)	.261	1.29(0.76,2.17)	.348
Q3	4.08(2.55,6.54)	< .001	2.42(1.45,4.04)	.001	2.08(1.18,3.68)	.012
Q4	6.72(4.31,10.46)	< .001	4.31(2.55,7.28)	< .001	3.15(1.72,5.78)	< .001
P for trend		< .001		< .001		< .001

Model 1: non-adjusted;

Model 2: adjusted for age, gender, race;

Model 3: adjusted for age, gender, race, educational level, marital status, PIR, smoking status, drinking status, BMI category, hypertension, diabetes, CVD, CKD, HDL-C, and TC.

RAR = red blood cell distribution width-to-albumin ratio; HUA = hyperuricemia; PIR = family poverty income ratio; BMI = body mass index; CVD = cardiovascular disease; CKD = chronic kidney disease; HDL-C = high-density lipoprotein cholesterol; TC = total cholesterol; HR = hazard ratio; CI = confidence interval.

Supplementary Table S3. Association between the red blood cell distribution width-to-albumin ratio and all-cause and cardiovascular mortality in adults with hyperuricemia after excluding participants aged 80 years or older.

Variables	Model 1		Model 2		Model 3	
	HR(95% CI)	*P*	HR(95% CI)	*P*	HR(95% CI)	*P*
**All-cause mortality**						
RAR	3.12(2.65,3.66)	< .001	2.55(2.06,3.16)	< .001	2.16(1.79,2.6)	< .001
RAR quartiles						
Q1	reference		reference		reference	
Q2	2.05(1.55,2.72)	< .001	1.64(1.24,2.15)	< .001	1.57(1.18,2.10)	.002
Q3	3.15(2.38,4.15)	< .001	2.20(1.63,2.97)	< .001	1.92(1.37,2.69)	< .001
Q4	6.67(5.10,8.73)	< .001	4.81(3.55,6.51)	< .001	3.62(2.61,5.01)	< .001
P for trend		< .001		< .001		< .001
**CVD mortality**						
RAR	3.48(2.82,4.28)	< .001	2.68(2.04,3.50)	< .001	2.38(1.79,3.15)	< .001
RAR quartiles						
Q1	reference		reference		reference	
Q2	1.56(0.87,2.81)	.138	1.29(0.71,2.32)	.401	1.34(0.72,2.51)	.361
Q3	2.97(1.61,5.47)	< .001	2.12(1.10,4.10)	.025	1.89(0.96,3.71)	.065
Q4	7.45(4.46,12.45)	< .001	5.55(3.06,10.07)	< .001	4.13(2.15,7.92)	< .001
P for trend		< .001		< .001		< .001

Model 1: non-adjusted;

Model 2: adjusted for age, gender, race;

Model 3: adjusted for age, gender, race, educational level, marital status, PIR, smoking status, drinking status, BMI category, hypertension, diabetes, CVD, CKD, HDL-C, and TC.

RAR = red blood cell distribution width-to-albumin ratio; HUA = hyperuricemia; PIR = family poverty income ratio; BMI = body mass index; CVD = cardiovascular disease; CKD = chronic kidney disease; HDL-C = high-density lipoprotein cholesterol; TC = total cholesterol; HR = hazard ratio; CI = confidence interval.
